# Structure–Activity Relationships in RuCs/MgO Catalysts During Ammonia Synthesis

**DOI:** 10.1002/cssc.202501035

**Published:** 2025-07-24

**Authors:** Linus Biffar, Niklas Martin Brinker, Peter Pfeifer, Roland Dittmeyer, Jan‐Dierk Grunwaldt, Dmitry E. Doronkin

**Affiliations:** ^1^ Institute for Micro Process Engineering Karlsruhe Institute of Technology Hermann‐von‐Helmholtz‐Platz 1 76344 Eggenstein‐Leopoldshafen Germany; ^2^ INERATEC GmbH Siemensallee 84 76187 Karlsruhe Germany; ^3^ Institute of Catalysis Research and Technology and Institute for Chemical Technology and Polymer Chemistry Karlsruhe Institute of Technology Hermann‐von‐Helmholtz‐Platz 1 76344 Eggenstein‐Leopoldshafen Germany

**Keywords:** ammonia synthesis, cesium, operando, ruthenium, X‐ray absorption spectroscopy

## Abstract

In the industrial synthesis of ammonia, a combination of high temperature and pressure is required to achieve a reasonable educt conversion. Efforts have been undertaken to lower these requirements by utilizing ruthenium‐based catalysts promoted with alkali metals, which have the potential to lower the energy barrier associated with the dissociative adsorption of nitrogen. In this work, the structure of Ru and Cs species is probed in impregnated RuCs/MgO and Ru/MgO catalysts by operando X‐ray absorption spectroscopy during reduction and ammonia synthesis at pressures up to 19 bar(a) in pure gas feed as well as the deactivation behavior with unpure feed containing 25 ppm oxygen. Interconversion of three types of Ru species, including RuO_2_, highly dispersed RuO_x_, and metallic Ru, occurs for both studied catalysts. Promotion by Cs leads to higher content of metallic Ru at the expense of dispersed RuO_x_ and results in higher NH_3_ concentration at the reactor outlet. Exposure of the catalysts to traces of oxygen enables a gradual transformation of bare Cs^+^ cations to hydrated species [Cs(H_2_O)_x_]^+^. The irreversible deactivation of the catalyst is traced back to the leaching of cesium species, which has a disproportionate effect on the catalytic activity.

## Graphical abstract

Ru and Cs species in RuCs/MgO catalysts are mapped under relevant temperatures and pressures of ammonia synthesis with the help of operando X‐ray absorption spectroscopy. Interconversion of three types of Ru (RuO_2_, highly dispersed RuO_x_ clusters, and metallic Ru) as well as hydrated and dehydrated Cs^+^ species are observed and correlated with ammonia production as a function of operation conditions.

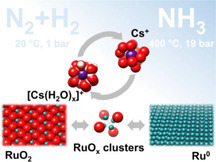

## 1 Introduction

Ammonia is one of the most important bulk chemicals, and the energy‐intensive synthesis accounts for 1%–2% of global energy consumption.^[^ ^1^ ^]^ As an additional emerging use case, green ammonia is considered to be a promising candidate for a carbon‐free chemical energy carrier, enabling the large‐scale transport of chemically bound hydrogen.^[^ ^2^, ^3^ ^]^ Traditionally, hydrogen for ammonia synthesis is produced via steam reforming of natural gas, which enables the stationary operation of the synthesis plant, while the production of green ammonia requires hydrogen produced by electrolysis. When operating any Power‐to‐X plant in conjunction with fluctuating renewable energies, the load flexibility of the plant becomes of great importance, as the cost of buffer tanks can quickly add up.^[^ ^4^, ^5^ ^]^ The synthesis of ammonia is enabled by catalysts; hence, the size, thermal inertia, and therefore the load modulation capabilities of the synthesis reactor are directly linked to the properties of the chosen catalyst.

The most commonly used catalysts in ammonia synthesis are bulk sintered Fe promoted with low amounts (approx. 1 wt%) of alkali and alkaline earth metals. Not only more active but also more expensive, Ru catalysts are usually supported on aluminum oxide, magnesium oxide, or activated carbon and use similar electronic promoters. The basics of the ammonia synthesis process are reasonably well understood.^[^ ^6^ ^]^ For iron catalysts, the effect of electronic promoters is proposed to be the polarization of the catalyst surface via electron donation to the conduction band of Fe, modifying the adsorption of N‐containing species. For cesium‐promoted Ru catalysts, two different mechanisms, electron donation from cesium hydroxide,^[^ ^7^ ^]^ and surface modification by cesium suboxide^[^ ^8^ ^]^ are proposed. The knowledge‐based development of ammonia synthesis catalysts requires an in‐depth understanding of the catalyst structure and functionality. To date, no study to uncover the structure of the active Ru and Cs species in RuCs catalysts under synthesis conditions has been undertaken due to the requirements of high temperature, high pressure, and reactive atmosphere, which are incompatible with commonly used surface‐sensitive techniques. Synchrotron‐based hard and tender X‐ray techniques are ideal for studying heterogeneous catalysts due to the penetrating power of X‐rays, which allows the use of in situ cells with varying gas, temperature, pressure, and gas flow patterns mimicking industrial conditions. Niemann, Clausen, and Topsøe have paved the way with operando X‐ray absorption spectroscopy (XAS) studies of Fe catalysts promoted with K, Rb, and Cs, finding both the influence of alkali metal promoters on the reducibility of Fe and also the fact of restructuring of said promoters without reduction under ammonia synthesis conditions.^[^ ^9^, ^10^ ^]^ However, given the complexity of the task and limitations of instrumentation in the late 1980s, no further information could be extracted on the state of alkali metal promoters.

Despite the scarcity of XAS studies on ammonia synthesis catalysts under working conditions, in situ XAS has also been applied to understand the structure of alkali metals in various materials. Shiota et al. investigated the stabilization of cesium in an alkali‐activated municipal solid waste incineration fly ash and pyrophyllite‐based system by in situ XAS at the Cs K edge and recorded spectra for multiple reference materials, which they used to perform principal component analysis (PCA) on the spectra of their sample.^[^ ^11^ ^]^ By choosing only two primary components, namely species with a Cs—O bond and pollucite, they were able to extract clear trends in the distribution of Cs species over the duration of their experiment. Utilizing the same technique on a different sample, it was also possible to identify a distinctly different reference (CsCl) as an additional third component with a significant contribution to the overall spectrum, but again, no further classification between species with a Cs—O bond could be made.^[^ ^12^ ^]^ Davies et al. performed temperature programmed oxidation of a K/Al_2_O_3_ catalyst while recording the potassium K edge and reported dehydration and decomposition of the precursor between 100–160 °C, and mobilization of the resulting potassium bicarbonate at 200–260 °C.^[^ ^13^ ^]^ Doskocil et al. studied the interaction of the alkali metal rubidium with various support materials at elevated temperatures in the presence of oxygen and showed that the choice of the support has a significant impact on the environment of the alkali metal, with Rb/MgO yielding the strongest basic sites.^[^ ^14^ ^]^

In this work, we investigate the structure and interaction of different Ru and Cs species by applying in situ and operando hard and tender XAS to monitor the respective species in unpromoted Ru/MgO and Cs‐promoted RuCs/MgO catalysts during the reductive activation and subsequent ammonia synthesis reaction. It has previously been reported that highly active RuCs/MgO catalysts tend to deactivate over time, and various mechanisms have been proposed.^[^ ^15^ ^]^ To narrow down the exact cause of this deactivation, we conducted experiments at elevated temperatures and with oxygen impurities in the feed gas, aiming to observe deactivation despite the limited time frame of synchrotron experiments.

## 2 Results and Discussion

High‐pressure operando XAS experiments were performed in a quartz capillary reactor^[^ ^16^ ^]^ operated at pressures of up to 19 bar(a) in pure and O_2_‐contaminated N_2_ + H_2_ feed while continuously recording XAS spectra around Cs K and Ru K adsorption edges for up to 14 h on stream per experiment. In addition, the reduction behavior of Ru and Cs was probed in temperature‐programmed reduction (TPR) experiments while recording Ru K and Cs L_3_ spectra. All measured XAS spectra were sorted by absorption edge (Ru K, Cs K, and Cs L_3_) and then imported as one large (>1000 spectra for K edges) dataset. Each dataset thus encompassed the combined spectra recorded across multiple different experiments for a selected absorption edge. Principal component analysis was performed on full datasets and suggested interconversion of three individual Ru and two individual Cs components throughout the measurement campaign. The spectra of the individual components and their respective fractions were extracted using the self‐modeling mixture analysis technique, multivariate curve resolution alternating least squares (MCR‐ALS).^[^ ^17^ ^]^ Analysis of all recorded spectra as combined datasets (i.e., spectra of unpromoted and promoted catalysts treated together) ensured the direct comparability of even small trends in the relative fractions of different species across different catalysts and experiments.

### 2.1 Identification of Ru Species

**Figure 1:**
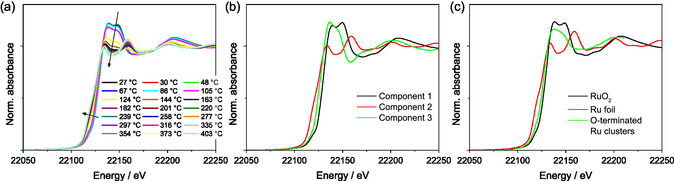
a) Normalized Ru K XANES spectra measured during TPR of the RuCs/MgO catalyst (averaging over 10 scans). b) Distinct spectral components identified via MCR‐ALS in the dataset containing all experimental Ru K XANES spectra. c) Reference spectra including RuO_2_ and Ru metal (foil) measured during the experiment, as well as a spectrum of oxygen‐terminated small Ru clusters (dispersed RuO_x_) extracted from ref. [21] and energy‐aligned using the spectrum of Ru foil reported in the same source.

Most of the spectral changes with respect to Ru species could be observed during reductive activation and TPR experiments; the selected Ru K edge X‐ray absorption near edge structure (XANES) spectra of RuCs/MgO recorded during TPR are given in **Figure** **1**a (Ru/MgO spectra are qualitatively similar). The three distinct spectral components obtained by analyzing the combined data of all Ru K edge measurements on both Ru/MgO and RuCs/MgO catalysts are reported in Figure 1 alongside reference spectra. Two components could be readily identified as RuO_2_ and metallic ruthenium. The third spectral component belongs to (partially) oxidized Ru species, as indicated by the rising edge position. However, the number and positions of peaks above the white line differ substantially from the RuO_2_ spectrum. This difference can not be attributed to the temperature effect, which mostly alters relative intensities.^[^ ^18^ ^]^ Furthermore, as will be shown below, the concentration profiles corresponding to (RuO_2_) and the unknown component do not follow the same trends, neither at low nor at high temperatures. The spectrum in question appears to be similar to the spectra reported as Ru single atom catalyst,^[^ ^19^ ^]^ and has also been reported in the Ru/MgO system as Ru single atoms.^[^ ^20^ ^]^ Zhu et al. analyzed the extended X‐ray absorption fine structure (EXAFS) region corresponding to the similar XANES component (taking into account different instrumentation and normalization) and found both Ru—O and Ru—Ru distances corresponding to oxygen‐terminated Ru clusters.^[^ ^21^ ^]^ In the following, this component will be referred to as dispersed RuO_x_.

XANES calculations^[^ ^22^ ^]^ were performed to exclude the possibility that the spectral component identified as dispersed RuO_x_ in fact represents surface Ru atoms in metallic Ru nanoparticles covered with chemisorbed oxygen and is not an individual species. Comparison of the distinct Ru spectral components with theoretically calculated Ru spectra is given in the supporting information (SI). While calculated peak intensities are typically unreliable, the features (i.e., number and especially the relative positions of peaks and valleys) in the calculated spectrum of oxygen‐terminated Ru clusters^[^ ^21^ ^]^ resemble the MCR‐ALS derived spectrum. In contrast, the spectrum of a surface Ru atom in a Ru nanoparticle terminated with surface O atoms still resembles the spectrum of bulk Ru metal, confirming that the intermediate Ru spectral component is unlikely to represent the surface of oxygen‐covered Ru nanoparticles.

### 2.2 Identification of Cs Species

**Figure 2:**
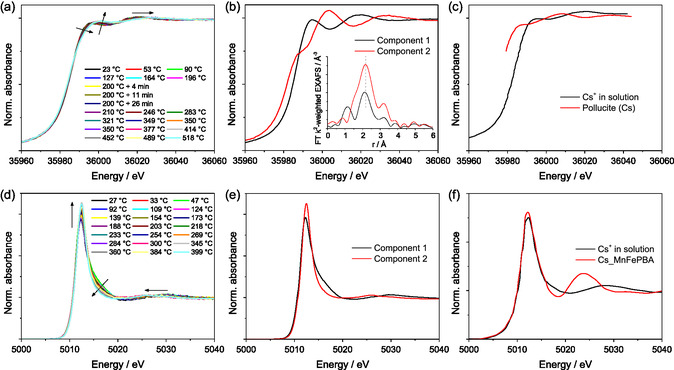
Normalized a) Cs K XANES and d) Cs L_3_ HERFD‐XANES spectra measured during (a) standard activation (averaging over each 6 scans) and (d) TPR of the RuCs/MgO catalyst (averaging over each 10 scans). Distinct spectral components identified via MCR‐ALS in the dataset containing all experimental Cs K XAS b) and Cs L_3_ HERFD‐XANES e) spectra. Inset in (b) shows the corresponding FT EXAFS spectra. Cs K c) and Cs L_3_ f) reference XANES spectra including Cs^+^ in solution and confined in the solid phase. Cs L_3_ XANES data comes from ref. [23] K edge data for Cs^+^ in solution is reported in ref. [24], and for pollucite in ref. [12].

Cs K and L_3_ edge XANES spectra obtained during (reductive) activation and TPR of the RuCs/MgO catalyst are shown in **Figure** **2**a,d. Two distinct spectral components were extracted from both the Cs K XAS and Cs L_3_ high‐energy resolution fluorescence detected (HERFD) XANES datasets, which are shown in Figure 2a,c, respectively. The smooth and stable background allowed MCR‐ALS to be performed in the full measured energy range. As a result, the Cs K edge spectral components allow extraction of the EXAFS signal (shown in the Supporting Information, SI), which was Fourier‐transformed (FT) and is shown in the inset of Figure 2a.

*k*‐space EXAFS clearly points at backscattering on light atoms as it is peaking below 5 Å^−1^ and the oscillations are already almost fully dampened at 8 Å^−1^. The first shell peaks are very broad, and further shells are not visible, highlighting disordered structures around Cs in both species. Component 1 (low temperature species) shows backscattering with a lower amplitude than component 2, possibly suggesting a lower coordination number but at higher distances (uncorrected for the phase shift).

As both Cs species are highly disordered, it is likely that the two extracted spectra do not necessarily resemble a single species but rather represent the combined spectra of multiple similar species (such as Cs^+^ with different numbers of H_2_O ligands). Moreover, meaningful spectral shapes and concentration profiles (albeit with significantly higher noise level) could also be obtained when assuming three spectral components, two of those occurring at high temperatures and having similar spectral shapes and trends in concentration profiles (provided in the SI). Therefore, discussing them as a single lumped species does not change the identification and allows more reliable quantification at the steady‐state, while describing the exact dehydration sequence of Cs^+^ species is out of the scope of the current work. This makes it unlikely that a perfect match to reference data can be found, but a rough classification should be possible by comparing the fingerprints of the isolated spectra to spectra of known Cs species. To identify the structure of the Cs species observed in the RuCs/MgO catalyst, similar reference spectra were found in the literature. Due to measurements being performed on different instruments with different energy resolution, an exact match is not possible, but relative positions (especially distances between the peaks) and relative intensities of spectral features (shoulders, peaks, and valleys) must match. For both K and L_3_ edge data, these features of each respective spectra of the low‐temperature component (component 1) fit well to the previously reported spectra of hydrated Cs^+^ (Cs^+^ in solution); this component will be referred to as [Cs(H_2_O)_x_]^+^.^[^ ^23^, ^24^ ^]^ Coordination numbers around hydrated Cs^+^ ions are reported to be in the range between 5 and 10, most commonly 6–8, and interatomic distances between Cs and O are in the range of 3.0–3.2 Å.^[^ ^25^ ^]^

The high‐temperature species (component 2) is more difficult to attribute. Cs K edge data resembles Cs species in clays and alumosilicates, with the best match being Cs‐containing zeolite mineral pollucite.^[^ ^12^ ^]^ Cs atoms in pollucite are coordinated by 12 oxygen atoms (6 at ≈3.4 Å and 6 at ≈3.6 Å)^[^ ^26^ ^]^ without any coordinated water molecules due to the lack of space in the zeolite cages. The corresponding Cs L_3_ spectrum matches a literature spectrum of Cs^+^ ions trapped in voids of Mn and Fe containing Prussian Blue analog (MnFePBA) structures,^[^ ^23^ ^]^ where, similar to the pollucite structure, Cs^+^ displaces water molecules from the voids they occupy due to space constraints. [Cs(H_2_O)_x_]^+^ would be too large to be included in the Prussian Blue analog (PBA) framework. The attribution of the high temperature species to pollucite‐like coordination of Cs^+^ with more oxygen neighbors located at longer distances, as compared to the low temperature hydrated [Cs(H_2_O)_x_]^+^, also agrees with the trends in coordination numbers and Cs—O distances observed in the FT EXAFS spectra. It has been observed that in some cases, such as potassium/perovskite systems, the promoter can be anchored into the support lattice.^[^ ^27^ ^]^ This is, however, highly unlikely in our system due to the size differences between Cs and Mg/O.

### 2.3 TPR

**Figure 3:**
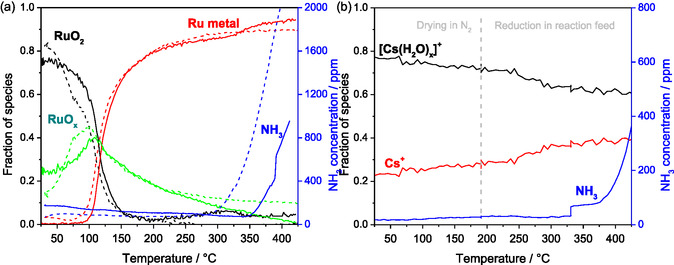
Evolution of spectral components obtained via MCR‐ALS analysis of a) Ru K edge XANES spectra recorded during TPR for both studied catalysts (solid lines stand for RuCs/MgO and dashed lines for Ru/MgO), b) Cs K edge XAS spectra during standard activation of the RuCs/MgO catalyst, including drying in N_2_ and reduction in the ammonia synthesis feed gas at 19 bar(a). Blue curves stand for ammonia concentration measured at the outlet of the in situ capillary cell.

To investigate the influence of the cesium promoter, a TPR of the unpromoted Ru/MgO and promoted RuCs/MgO catalysts was conducted. In three separate experiments, the fractions of each ruthenium and cesium species were determined and plotted over the temperature together with the concentration of the produced ammonia (**Figure** **3**). Measurements of cesium were conducted during standard catalyst activation, as no significant reduction of Cs was observed during ammonia synthesis experiments. At 330 °C, the temperature ramp was paused for 20 min to record multiple spectra for averaging, which explains the small jump in outlet NH_3_ concentration at this temperature.

As synthesized, ruthenium is mostly in the oxidized state as bulk crystalline RuO_2_ in both catalysts, and nearly no metallic ruthenium can be detected. The reduction starts at 50 °C for the unpromoted catalyst and at around 80 °C for the cesium‐promoted catalyst. Up to 105 °C, mostly oxygen‐terminated small RuO_x_ clusters (dispersed RuO_x_) are formed, which during further reduction agglomerate to metallic ruthenium. The dispersion of RuO_2_ may be facilitated by interaction of this acidic oxide with basic MgO support,^[^ ^28^ ^]^ this effect has previously been exploited to prepare “single‐atom” and highly dispersed Ru/MgO catalysts.^[^ ^20^, ^29^ ^]^ Preferred interaction of RuO_2_ with more basic Cs species would also explain its slower dispersion in the Cs‐promoted catalyst. At a temperature of 200 °C, the reduction is mostly complete, and some differences between the catalysts become apparent. With the unpromoted catalyst, gradual continuous reduction of ruthenium proceeds at a low rate, and at 300 °C no RuO_2_ is detected. As the produced ammonia also emerges at this temperature, this leads to the assumption that the active sites relevant for the dissociative adsorption of nitrogen have a strong affinity to oxygen and are reduced to metallic ruthenium last. These sites are believed to be specifically the B_5_ sites on metallic Ru particles.^[^ ^30^ ^]^

In contrast to this, a plateau with a slight increase in oxidized ruthenium is observed between 200 and 320 °C with the promoted catalyst, while no ammonia is formed. Larichev et al. showed that a thin layer with a thickness of less than 1 nm of a disordered structure was present on the ruthenium metal particles, the diffraction pattern of which in the as‐received catalyst was similar to that of cesium hydrocarbonate CsHCO_3_.^[^ ^31^ ^]^ We, however, under all conditions also observed the hydrated [Cs(H_2_O)_x_]^+^ species, which were previously not reported due to the impossibility to observe disordered species by the used X‐ray diffraction technique and/or different sample storage conditions, which are typically not described in the cited works. Independent of their exact nature, both observed species probably act as a strong base and cover parts of the ruthenium clusters, thus inhibiting the reduction process.

In a temperature range between 250 and 300 °C, a continuous increase in the fraction of Cs^+^ species is observed, which indicates a gradual conversion from the hydrated [Cs(H_2_O)_x_]^+^ species. Larichev et al. ascribed the Cs species in the reduced catalyst to the suboxide Cs_2+x_O,^[^ ^8^, ^32^ ^]^ while Aika et al. assigned them to cesium hydroxide CsOH,^[^ ^7^, ^33^ ^]^ both groups basing their conclusions on X‐ray photoelectron spectroscopic (XPS) data. Neither EXAFS nor XANES experimental spectrum of high temperature species observed by us in Figure 2b suggest reduction of Cs species and the pollucite spectrum has a similar shape of the rising edge and white line (but not the EXAFS region) compared to spectra of anhydrous CsOH and CsHCO_3_,^[^ ^12^ ^]^ i.e., the same electronic state as observed in the XPS studies by Aika et al.^[^ ^7^, ^33^ ^]^ The disordered nature of Cs^+^ observed by us would also explain the lack of exact match of binding energies between the respective catalyst and the reference CsOH observed in the previous XPS studies.^[^ ^33^ ^]^

At about 320 °C an increase of metallic ruthenium and a decrease of remaining ruthenium oxide fractions are observed. This occurs without an increase in the relative fraction of noncrystalline surface oxidized RuO_x_ clusters. The trend could also be explained by Cs species preferentially covering ruthenium oxide surfaces. The ongoing dehydration of Cs species (Figure 3b) therefore, enables previously protected ruthenium oxides to be reduced to metallic ruthenium. After the reduction of the remaining ruthenium sites, the catalyst starts to show activity at 350 °C, again indicating that the adsorption sites responsible for the synthesis of ammonia have a strong affinity for oxygen and are reduced last.

Highly dispersed surface oxidized ruthenium clusters RuO_x_ continue to agglomerate to larger metallic particles on the cesium‐promoted catalyst until the end of the TPR. Contrary to this, with the unpromoted catalyst, the distribution of ruthenium species seems to stabilize above 350 °C. This implies that cesium species stabilize metallic ruthenium and/or increase the mobility of noncrystalline dispersed ruthenium clusters on the catalyst surface. After the TPR, more metallic ruthenium is present on the promoted catalyst, which is in agreement with observations by Larichev, where this was explained by the presence of Cs^+^ ions promoting the reduction of ruthenium.^[^ ^32^ ^]^ In contrast to this, we identified noncrystalline dispersed ruthenium clusters as the third species and showed that the higher fraction of metallic ruthenium in the promoted catalyst correlates with the lower fraction of noncrystalline dispersed ruthenium. The observation may be explained by the interaction of Cs species with the magnesia support, which blocks nucleation sites of the support and thus limits the dispersion of ruthenium, forcing it to form larger and easier‐to‐reduce nanoparticles.^[^ ^32^, ^33^ ^]^ Furthermore, no ruthenium oxide was detected in the unpromoted catalyst. Our experiments show that not only are rather isolated Cs^+^ ions present at the end of the reduction, but also hydrated cesium [Cs(H_2_O)_x_]^+^, which possibly prevents a complete reduction of ruthenium.

### 2.4 Steady‐State Ammonia Synthesis with and without Oxygen Impurities

**Figure 4:**
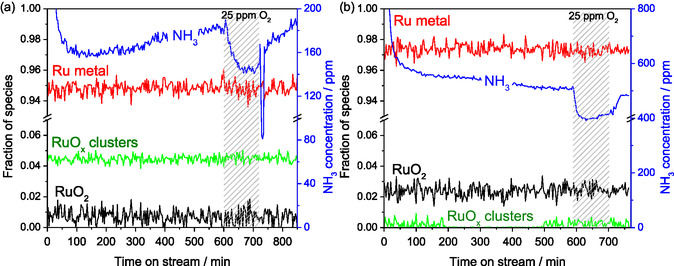
Evolution of Ru spectral components during NH_3_ synthesis at 19 bar(a) and 386 °C with time on stream in pure feed, during additional dosing of 25 ppm O_2_ impurity (marked as a gray pattern fill), and after subsequent switch to pure stream over a) Ru/MgO and b) RuCs/MgO catalyst. Blue curves stand for ammonia concentration at the outlet of the in situ capillary cell.

To investigate the effect of the promoter on the distribution of the individual ruthenium and cesium species during ammonia synthesis, an operating point was measured at 19 bar(a) and 386 °C with 20 NmL min^−1^ and a stochiometric ratio of H_2_/N_2_ = 3 for 12 h. After about 9 h, educt gas containing 25 ppm oxygen was dosed to examine the inhibition of the reaction rate as well as the subsequent regeneration when switching back to an oxygen‐free feed. The fractions of individual ruthenium species, as well as the ammonia signal recorded by the Fourier transforminfrared spectrometer (FTIR) gas analyser, are presented in **Figure** **4**. No change in the distribution of ruthenium species is observed in both experiments during ammonia synthesis in pure gas feed. Figure 4a shows the results of the unpromoted Ru/MgO catalyst, and a slight increase in activity is observed despite the seemingly complete reduction. After changing to the oxygen‐containing feed gas, the catalytic activity decreases but is fully restored after switching back to the oxygen‐free feed. The catalyst itself, therefore, does not irreversibly deactivate, but the reaction is inhibited by oxygenates occupying active adsorption sites. As no oxidation of ruthenium is observed, it is possible that the small increase in activity at the beginning of the experiment can also be explained by the desorption of remnant adsorbed oxygenates. The experiment was repeated with a promoted RuCs/MgO catalyst. In Figure 4b, a significantly increased activity in comparison to the unpromoted catalyst is observed, which then steadily decreases over time. After switching to the oxygen‐containing feed gas, the catalytic activity drops by a similar relative amount as with the unpromoted catalyst. Again, this effect is mostly reversed when switching back to an oxygen‐free feed gas. When extrapolated until the end of the experiment, the catalytic activity continues to slowly decline according to the same trend as prior to the exposure to oxygen‐containing feed gas. This suggests that the rate of irreversible deactivation of the promoted RuCs/MgO catalyst is independent of oxygen in the feed gas at low concentrations.

Operando XANES and EXAFS spectra measured at Ru K edge during ammonia synthesis in pure feed at 19 bar(a) and 386 °C, average of all spectra measured under the same conditions, are presented in the SI. The same set of XANES spectral features located at the same energies for the bulk Ru metal and the RuCs/MgO catalyst (Figure S6a, Supporting Information) does not support the hypothesis of electronic interaction between Cs and Ru species. The corresponding EXAFS analysis (Table S1, Supporting Information) with the assumption of a Gaussian pair distribution function^[^ ^34^ ^]^ allows to estimate the first shell Ru—Ru coordination numbers in the Ru/MgO catalyst as 9.0 and RuCs/MgO as 9.6, which are comparable and do not contradict the hypothesis that Cs promoter limits dispersion of Ru species. The coordination numbers are obtained using standard EXAFS formalism, assuming harmonic thermal vibrations, which is not the case for nanoparticles at high temperatures.^[^ ^34^ ^]^ This leads to significant underestimation of coordination numbers and, therefore, particle sizes.^[^ ^34^ ^]^ To obtain true particle sizes in this case, EXAFS analysis should be based on the pair distribution function obtained via molecular dynamics simulations, which is outside the scope of this work.

**Figure 5:**
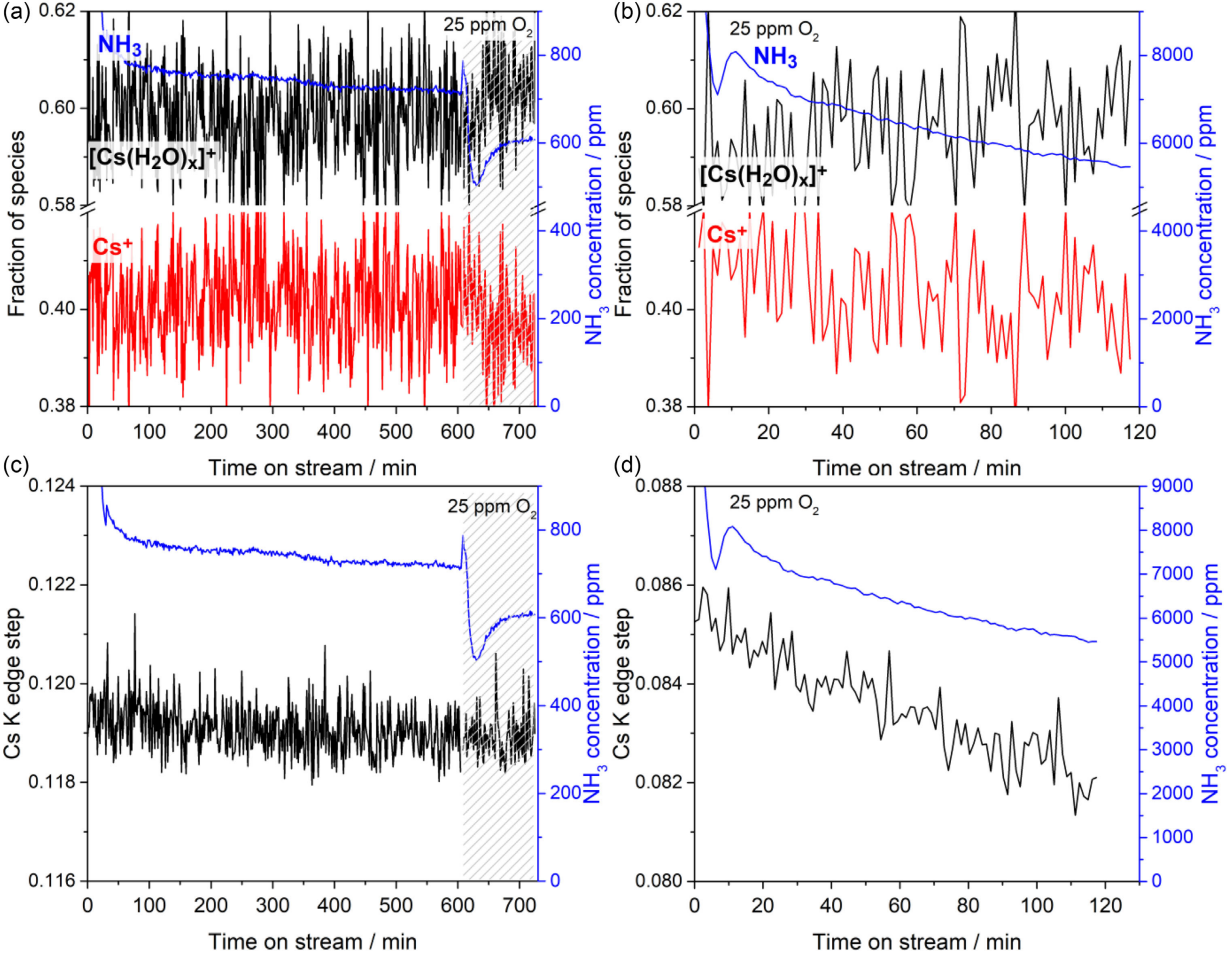
Evolution of a) Cs spectral components and c) absolute height of Cs K edge (“edge step”) for the RuCs/MgO catalyst during NH_3_ synthesis at 19 bar(a) and 386 °C with time on stream in pure feed and during additional dosing of 25 ppm O_2_ impurity (marked as a gray pattern fill). b,d): the corresponding data (on a new sample) measured at 19 bar(a) and 490 °C in the NH_3_ synthesis feed containing 25 ppm O_2_. Blue curves stand for ammonia concentration at the outlet of the in situ capillary cell.

The same experiment was repeated, this time measuring the Cs K absorption edge (**Figure** **5**). To accelerate the deactivation, experiments at higher temperatures of 490 °C (Figure 5b and S4, Supporting Information) and 686 °C (Figure S5, Supporting Information) were also conducted. In addition to the distribution of individual cesium species, the absolute height of the cesium absorption edge, the so‐called “edge step”, which correlates with the total cesium content of the sample, is plotted (Figure 5c,d). Analogously to Figure 4, a relatively slow but steady decrease in activity is observed at 386 °C in Figure 5a, with no clear trends in the distribution of both cesium species. Inhibition of the reaction and a slight shift toward the hydrated [Cs(H_2_O)_x_]^+^ species are observed after dosing oxygen‐containing feed gas. After switching back to oxygen‐free feed gas, the in situ capillary reactor cell broke, so no further data could be collected during the regeneration phase. Even though the individual cesium species did not change significantly during the experiment, the height of the absorption edge shows a slight downward trend during the whole experiment, with no significant change when exposed to the oxygen‐containing feed gas (Figure 5c). The steadily decreasing concentration of Cs in the probed catalyst volume supports the conclusion that oxygen in the feed gas is not the main cause for the irreversible deactivation of the catalyst, as it was previously assumed.^[^ ^15^, ^35^ ^]^

**Scheme 1:**
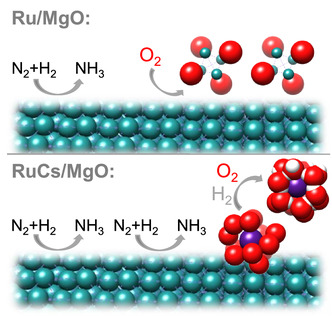
Illustration of Ru and Cs species relevant for ammonia synthesis in unpure feeds over Ru/MgO and RuCs/MgO catalysts.

This deactivation is more pronounced at a higher temperature of 490 °C (Figure 5b). While the operating point could only be recorded for 2 h due to time constraints at the beamline, the loss of catalytic activity was significantly accelerated, and the height of the cesium absorption edge shows a clear decreasing trend while the total absorbance background, i.e., sample density, stays relatively constant (Figure 5d). Again, a slight shift toward the hydrated [Cs(H_2_O)_x_]^+^ species can be observed (Figure 5b). As this was the predominant species before the reduction of the catalyst, the conversion from the hydrated species to the isolated Cs^+^ species is reversible to a certain degree in the presence of oxygen. As the catalytic activity drops by over 30% in Figure 5d, the height of the absorption edge disproportionately decreases by only about 4%. This suggests that the leaching Cs species are most likely the same Cs species that contribute to the improved catalytic activity of the catalyst. Two explanations for this improved catalytic activity of the cesium‐promoted catalyst are discussed in the literature. Aika et al. suggest that the ability of cesium to transfer electrons to the ruthenium surface from species such as CsOH or Cs_2_O, due to its low electronegativity, accelerates the dissociative adsorption of nitrogen.^[^ ^7^, ^33^ ^]^ Larichev et al. proposed that Cs^+^ cations form a thin film of cesium suboxide Cs_2+x_O covering the metallic ruthenium surface and argued that this leads to a decrease of the electron work function by polarizing effects, therefore facilitating the dissociation of nitrogen.^[^ ^31^, ^36^ ^]^ As the relatively slow leaching of Cs has a disproportionately large effect on the catalytic activity, we conclude that it is more likely that the thin film of dehydrated cesium species is responsible for the high activity of the active sites, while hydrated cesium species act more like a structural promoter stabilizing the metallic ruthenium (**Scheme** **1**).

## 3 Conclusion

The structure of Cs species and their influence on the dynamics and structure of Ru in unpromoted Ru/MgO and promoted RuCs/MgO catalysts were studied by operando XAS on a relatively long timescale during the synthesis of ammonia under industrially relevant conditions at elevated pressure and temperature. By combining large datasets and employing chemometric tools such as MCR‐ALS, even small structural changes could be observed. It was found that in a pure H_2_/N_2_ feed, the Cs promoter significantly dampens the formation of dispersed RuO_x_ clusters, resulting in a higher fraction of fully reduced metallic Ru surface (Scheme 1). The observed dampening of the intermediate RuO_x_ clusters may also contribute to an improved resistance of Ru particles toward sintering via the Ostwald ripening mechanism.^[^ ^37^ ^]^

Cesium, in the form of hydrated [Cs(H_2_O)_x_]^+^, covers RuO_2_ species in the as‐received RuCs/MgO catalyst. The hydrated Cs species are partially dehydrated during heating in the reaction feed gas, in agreement with thermodynamic equilibrium calculated in the SI using data from ref. [38] The ammonia synthesis reaction only starts when the reduction of ruthenium is nearly complete, showing that the adsorption sites responsible for the dissociative adsorption of nitrogen have a strong affinity for oxygen. While the structure and concentration of Ru species do not change significantly during long‐term experiments in the pure feed and feed contaminated with 25 ppm O_2_, Cs^+^ species can scavenge oxygen and store it in the coordination shell as water ligands. The inhibition by O_2_ impurities occurs for both unpromoted and promoted catalysts, possibly via blocking active Ru sites. This inhibition can be reversed by switching to pure H_2_/N_2_ feeds.

Irreversible deactivation was observed for the otherwise more active RuCs/MgO catalyst. The rate of deactivation did not depend on the presence of O_2_ impurities but rather on the reaction temperature. A steady leaching of cesium was identified by the gradual decrease of the Cs absorption edge step while the total absorbance of the sample remained constant, with faster leaching observed at higher temperatures. The leached cesium species are most likely also the ones most responsible for the catalytic activity enhancement, as the decrease in catalytic activity is not proportional to the leaching of cesium.

## 4 Experimental Section

4.1

4.1.1

##### Catalyst Synthesis

The unpromoted Ru/MgO and promoted RuCs/MgO catalysts were prepared via wet impregnation with ruthenium loading of 2 wt% and a molar ratio of Cs/Ru = 1 for the promoted catalyst. Magnesium oxide (MgO, Sintermagnesia, 200 Mesh, Magnesia GmbH) was crushed down to a Sauter‐diameter of about 2.5 μm via wet milling in ethanol. The resulting suspension was diluted with ethanol to a concentration of 50 mg mL^−1^. An appropriate amount of ruthenium‐acetylacetonate (Ru−acac, >97 % purity, Sigma‐Aldrich) was dissolved in the suspension, which was subsequently stirred for 4 h. The solvent was then evaporated in a rotary evaporator at a temperature of 60 °C and a pressure of 200 mbar. To decompose the acetylacetonate precursor, the dark‐red powder was placed in a muffle furnace at 170 °C for 5 h under ambient atmosphere, resulting in the Ru/MgO catalyst. To synthesize the promoted catalyst RuCs/MgO, the Ru/MgO agglomerates were crushed in a ball mill. Cesium carbonate (Cs_2_CO_3_, >99% purity, Sigma‐Aldrich) was dissolved in ethanol at a concentration of about 5 mg mL^−1^, and the Ru/MgO powder was added. After stirring the suspension for 4 h, the ethanol was again removed in a rotary evaporator. The catalyst was then pelletized, crushed, and sieved to a fraction between 50–100 μm. Prior to the measurements, the catalysts were stored in ambient air.

##### Hard X‐ray (Ru K and Cs K Edges) Operando XAS Measurements

Hard X‐ray absorption spectroscopic measurements were performed at the BM23 beamline of the European synchrotron radiation facility (ESRF) in transmission mode using ionization chambers as detectors.^[^ ^39^ ^]^ A double crystal monochromator (DCM) with Si(111) and Si(311) crystal pairs was used to filter the energy of incident X‐rays around the Ru K and Cs K edges, respectively. Rh and Pt‐coated mirrors were used to reject higher harmonics, and the beam size was set using slits to 0.3 mm (vertical) × 2 mm (horizontal), centered in the middle of the sample. Ru foil and a Xe gas‐filled chamber were used as standard samples for energy calibration.

For each experiment, about 12–15 mg of catalyst was fixed in a glass capillary (diameter 1.7–2 mm, wall thickness 0.02 mm, WJM ‐ Glas Müller GmbH), with quartz wool. The capillary was fixed and sealed with epoxy glue (Loctite 3450) in a stainless‐steel sample holder connected to the gas dosing setup, back pressure regulator, dilution flow, and FTIR gas analytics. Gas was dosed via calibrated mass flow controllers (Bronkhorst EL‐Flow), and the system pressure was controlled with an electronic back‐pressure regulator (Bronkhorst EL‐Press). Gas feeds were mixed from pure nitrogen (Purity 6.0, AIR PRODUCTS, FR) or nitrogen with 100 ppm oxygen (AIR PRODUCTS, FR) and hydrogen (Purity 5.2, GASIN, FR). The temperature of the capillary was controlled with a hot air blower (FMB Oxford GSB‐1300), and the temperature calibration was additionally checked using a 0.3 mm K‐type thermocouple touching the capillary below and above the catalyst sample. After the back pressure controller, the product gas was diluted with a 479 mL min^−1^ flow of nitrogen and analyzed with an FTIR gas analyzer (Gasmet DX4000).

Two testing protocols were used in the study, each time starting with a new capillary loaded with a fresh catalyst sample. To evaluate the reducibility of Ru species, TPR was performed at a temperature ramp of 2 K min^−1^ in a flow of 50 mL min^−1^ of 10% H_2_ in N_2_ at ambient pressure. For the ammonia synthesis reaction, the catalyst samples were first dried at 200 °C (ramp to setpoint 5 °C min^−1^) for 30 min in N_2_ flow at ambient pressure. Next, the catalyst samples were reduced in a 3:1 (stoichiometric) ratio of H_2_ and N_2_ with a total flow of 40 mL min^−1^ at 19 bar(a) with a temperature ramp of 5 K min^−1^ to 490 °C. Subsequently, the total gas feed flow was decreased to 20 mL min^−1^ and the temperature was lowered to 386 °C, unless specified otherwise, for long‐term deactivation and reactivation experiments. The reported ammonia concentration was corrected with respect to the dilution flow to represent concentration at the outlet of the in situ cell. Note that such a correction also amplifies the background signal, which is mainly due to ammonia sticking to the inner surfaces of components, predominantly tubing and fittings, since very long flushing periods are impractical during a synchrotron experiment.

##### Tender X‐ray (Cs L_3_ Edge) Measurements

TPR of the RuCs/MgO catalyst was additionally performed in the tender X‐ray spectrometer^[^ ^40^ ^]^ of the ESRF ID26 beamline while recording HERFD‐XANES spectra at the Cs L_3_ edge. For this purpose, the energy of the incident X‐rays was filtered by a Si(111) DCM, while X‐ray fluorescence was directed to Si(311) analyzer crystals tuned to the maximum of the Cs Lα_1_ emission line, which was recorded using a Dectris PILATUS3 X 100 K‐M detector and normalized per counts of a diode recording X‐rays scattered on a polyimide foil placed in a beam path before the sample compartment.

RuCs/MgO was loaded in a specially designed stainless steel cell covered with a 50 μm thick polyimide film and sealed with 200 μm thick graphite seals.^[^ ^41^ ^]^ TPR procedure was analogous to the hard X‐ray experiment with the exception of the maximum temperature not exceeding 400 °C due to limitations imposed by the thermal stability of the used polyimide window.

##### XAS Data Analysis

All measured hard X‐ray spectra^[^ ^42^ ^]^ were batch converted from HDF5 to ASCII format using BM23 data extraction scripts based on h5py library, sorted by absorption edge (Ru K and Cs K) and then, after rejecting incomplete scans, imported into Fastosh v.1.0.7 2nd prerelease software^[^ ^43^ ^]^ as one large (>1000 spectra) dataset per each absorption edge (i.e., Ru K edge data on both promoted and unpromoted sample treated together). The energy scale was calibrated and aligned using spectra of Ru foil and Xe gas reference samples. The XANES spectra were normalized using the Larch approach with post‐edge region between 50 and 600 eV for the Ru K dataset and with default parameters for the Cs data. For further analysis, the following ranges were selected: 21 913–22 245 eV for the Ru K data and 35 623–36 584 eV for the Cs K data. Next, PCA was performed, which suggested interconversion of three individual Ru and two individual Cs components in the respective datasets. The spectra of the individual components and their respective fractions were extracted using the MCR‐ALS toolbox^[^ ^17^ ^]^ built in the Fastosh software. Care should be taken when analyzing data measured at different temperatures together, as temperature may also affect the shape of XANES spectra,^[^ ^18^ ^]^ hence, minor spectral differences are discussed only in the context of long‐term measurements performed at the same temperature. The minimum number of species to resolve were set to three in the Ru K dataset, as it clearly showed no isosbestic points for at least parts of the spectral region (e.g. 22 150–22 160 eV), and two for the Cs datasets since identification of isosbestic points was complicated due to high noise in the data. Evolving factor analysis was used for the initial estimation of concentration profiles. Simple‐to‐use interactive self‐modeling mixture analysis (SIMPLISMA) approach was also tried for the initial estimation of spectra and led to similar end results. Non‐negativity and closure constraints were applied to all pure coefficients; only the non‐negativity constraint was applied to the spectra.

Attempts to resolve more than three/two individual components with MCR‐ALS were made but resulted in not physically meaningful spectral shapes (e.g., absorbance decreasing to zero above the absorption edge or, in general, not based on a sigmoid function) and/or two or more concentration profiles following the same trend. Especially in the case of Cs data, precise determination of the number of pure spectral components is difficult since three spectral components also result in meaningful concentration profiles and spectral shapes. However, in this case, the spectra and concentration profiles of two high‐temperature species are very similar to each other, possibly representing different degrees of hydration; hence to reduce the uncertainties and the noise level, it was decided to treat high‐temperature species as one lumped component.

Cs K spectral components extracted with MCR‐ALS were further transformed into *k*‐space, *k* ^*2*^‐weighted, multiplied by the Hanning window with the range 2–8 Å^−1^ and sill size 1 Å^−1^, and Fourier transformed in Fastosh. The data was not corrected for the phase shift.

Cs L_3_ HERFD‐XANES spectra^[^ ^44^ ^]^ were manually sorted to exclude faulty spectra and spectra with glitches (e.g., due to failures of the scan engine or the detection system); each five spectra were averaged and exported as ASCII files using pyMCA v.5.8.7 software.^[^ ^45^ ^]^ The resulting averaged spectra were imported into Fastosh, truncated after 5097 eV, and normalized in the same way as the hard X‐ray spectra. The dataset was deconvoluted into individual components using MCR‐ALS applied in the range 5000–5040 eV.

For all measured edges, treatment of all measured spectra per one edge (i.e., Ru K edge data for both catalysts lumped together) as one single dataset allows one to ensure direct comparability of trends in the relative fractions of different species across different catalysts and experiments.

## Conflict of Interest

The authors declare no conflict of interest.

## Supporting information

Supplementary Material

## Data Availability

The data that support the findings of this study are openly available in [ESRF] at [https://doi.org/10.15151/ESRF‐ES‐1368347815], reference number [1368347815].
