# Supplementary material for: Structure–Activity Relationships in RuCs/MgO Catalysts During Ammonia Synthesis
Source: ChemSusChem. 2025 Jul 24;18(19):e202501035. doi: 10.1002/cssc.202501035 (PMC12487743; doi:10.1002/cssc.202501035)
Supplement: Supplementary file 1 — Supplementary Material [file CSSC-18-e202501035-s001.pdf]

Supporting Information

Structure – Activity Relationships in RuCs/MgO Catalysts during Ammonia Synthesis

Linus Biffar[a], Niklas Martin Brinker[a], Peter Pfeifer [a,b], Roland Dittmeyer[a], Jan-Dierk Grunwaldt[c], Dmitry E. Doronkin[c]*

[a]Institute for Micro Process Engineering, Karlsruhe Institute of Technology,

Hermann-von-Helmholtz-Platz 1, Eggenstein-Leopoldshafen, 76344, Germany

[b]INERATEC GmbH, Siemensallee 84, Karlsruhe, 76187, Germany

[c]Institute of Catalysis Research and Technology and Institute for Chemical Technology and

Polymer Chemistry, Karlsruhe Institute of Technology,

Kaiserstr. 12, Karlsruhe, 76131, Germany

*e-mail: dmitry.doronkin@kit.edu

Contents

SI-1: Comparison of MCR-ALS-resolved spectra of individual Ru species and the corresponding theoretically calculated spectra 3

SI-2: MCR-ALS resolved Cs K and L_3 edge data assuming three spectral components 4

SI-3: Temperature-Programmed Reduction of RuCs/MgO measured at Cs L_3 edge based on the assumption of two spectral components 5

SI-4: *k*-space EXAFS data for individual Cs K spectral components 6

SI-5: Concentrations of Ru and Cs species during ammonia synthesis at higher temperatures 7

SI-6: Calculated temperature dependence of equilibrium concentrations of Cs cations coordinated with 1 to 4 water ligands 9

SI-7: Ru K edge XANES, EXAFS spectra of the Ru/MgO and RuCs/MgO, and the corresponding EXAFS analysis 10

References: 11

SI-8: FEFF.inp input files for the XANES calculations 12

SI-1: Comparison of MCR-ALS-resolved spectra of individual Ru species and the corresponding theoretically calculated spectra

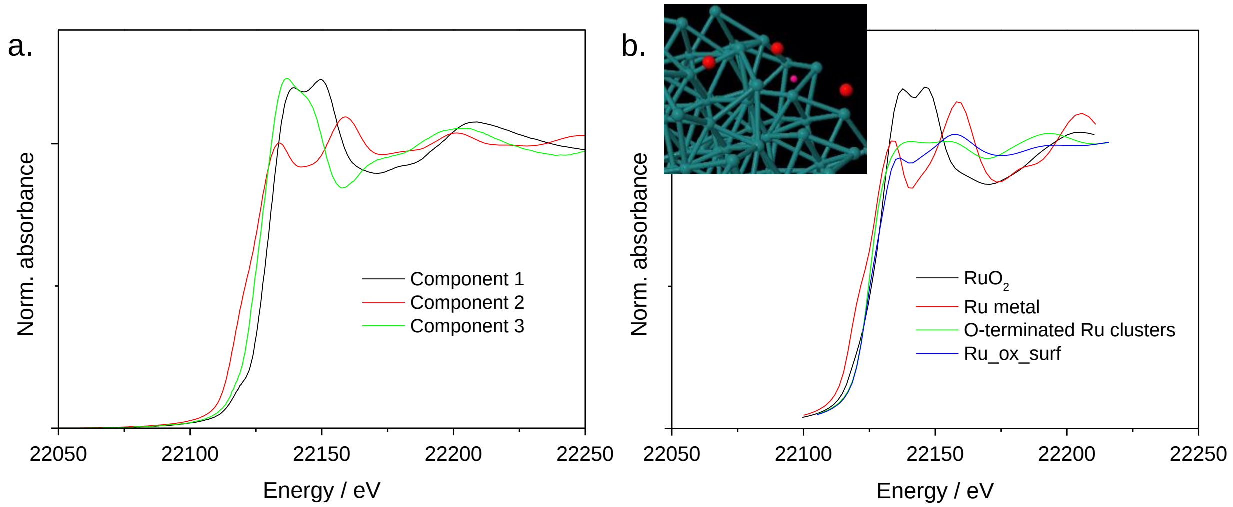

Figure S1: a. Distinct spectral components identified via MCR-ALS in the dataset containing all experimental Ru K XANES spectra (without additional normalization after MCR-ALS). b. Theoretically calculated XANES spectra (inset shows the surface Ru atom (pink) in vicinity of oxygen neighbors, for which the spectrum denoted "Ru_ox_surf" was calculated).

Calculations were performed using FEFF10[1] based on bulk Ru metal and RuO_2 structures (ICSD collection codes 43710 and 15071). Ru_4O_4 cluster structure were obtained from the bulk Ru structure by cutting out a single Ru_4 tetrahedron, adding four O atoms above the Ru_3 planes and optimizing geometry in Avogadro v.1.2.0. Partially oxidized surface Ru atom was modeled starting from a 1.4 nm Ru cluster with three oxygen atoms placed next to three topmost Ru atoms, following geometry optimization in Avogadro v.1.2.0. The Ru atom whose spectrum is calculated is shown in pink, neighboring O atoms in red (inset in Fig. S1b). FEFF.inp files are provided at the end of the supporting information.

SI-2: MCR-ALS resolved Cs K and L_3 edge data assuming three spectral components

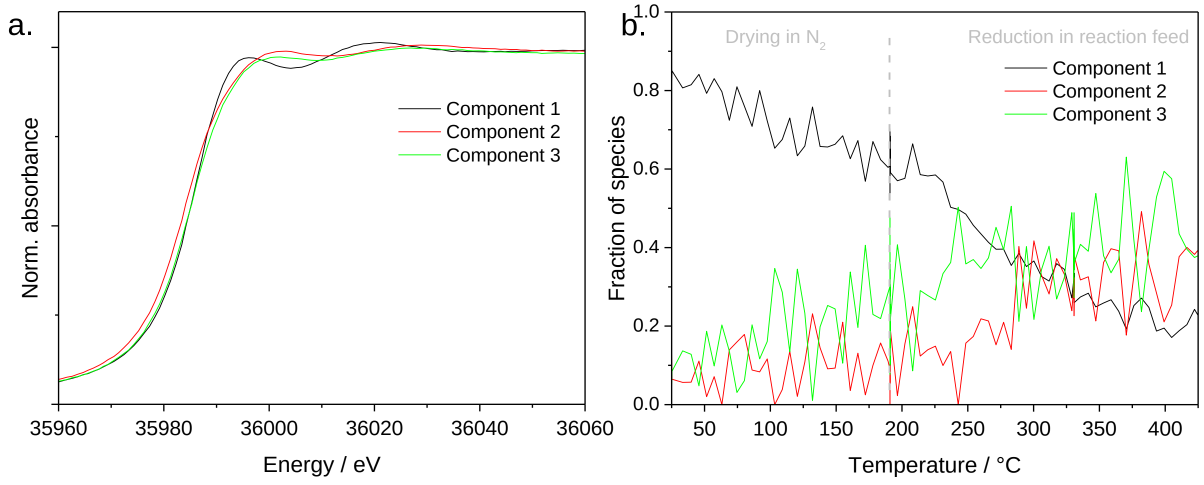

Figure S2: Three distinct spectral components and the corresponding fractions obtained via MCR-ALS analysis of Cs K XANES dataset measured during standard activation of the RuCs/MgO catalyst including drying in N_2 and reduction in the ammonia synthesis feed gas at 19 bar(a).

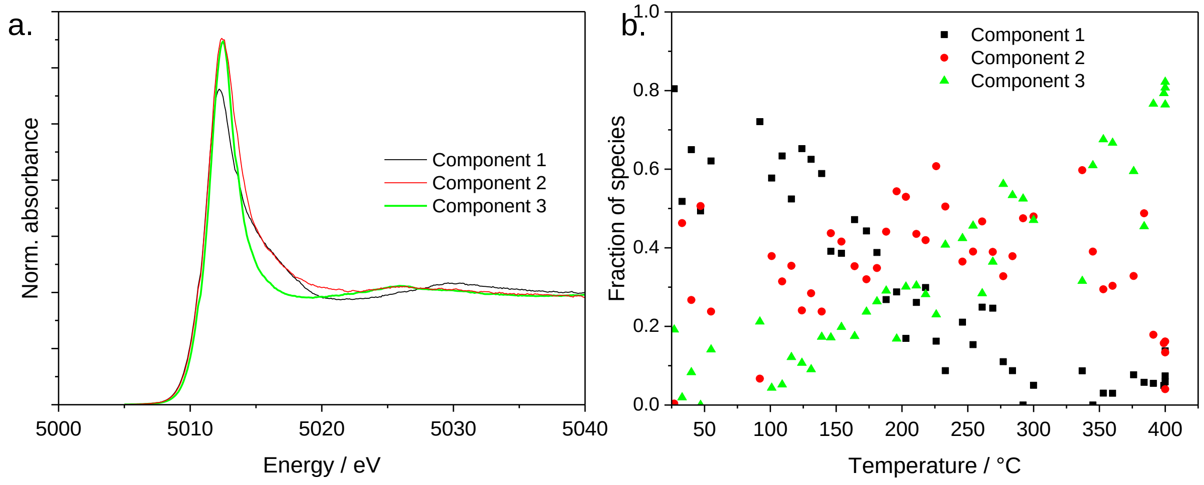

Figure S3: Three distinct spectral components and the corresponding fractions obtained via MCR-ALS analysis of Cs L_3 HERFD-XANES dataset measured during TPR of RuCs/MgO.

SI-3: Temperature-Programmed Reduction of RuCs/MgO measured at Cs L_3 edge based on the assumption of two spectral components

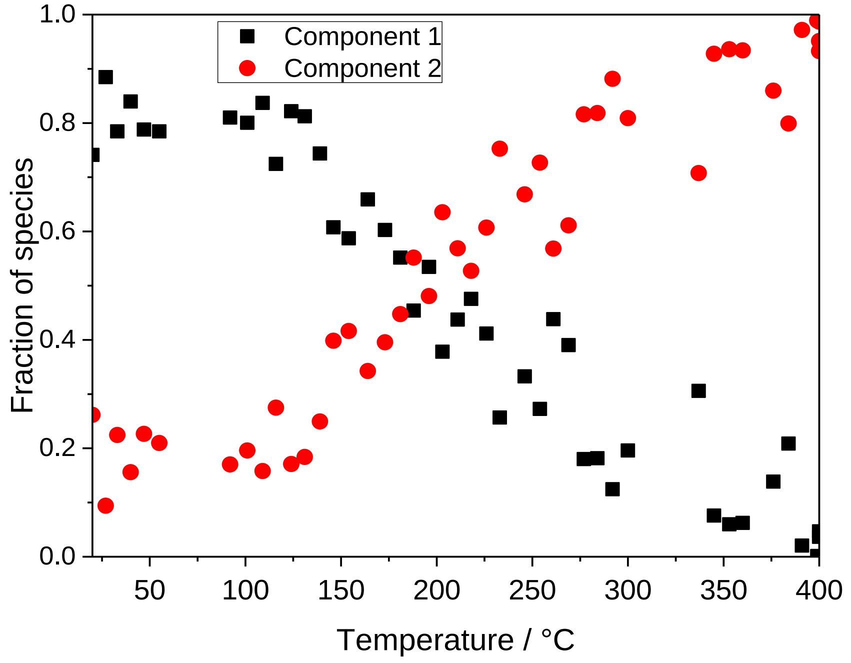

Figure S4: Evolution of spectral components obtained via MCR-ALS analysis of Cs L_3 HERFD-XANES dataset measured during TPR of RuCs/MgO.

SI-4: *k*-space EXAFS data for individual Cs K spectral components

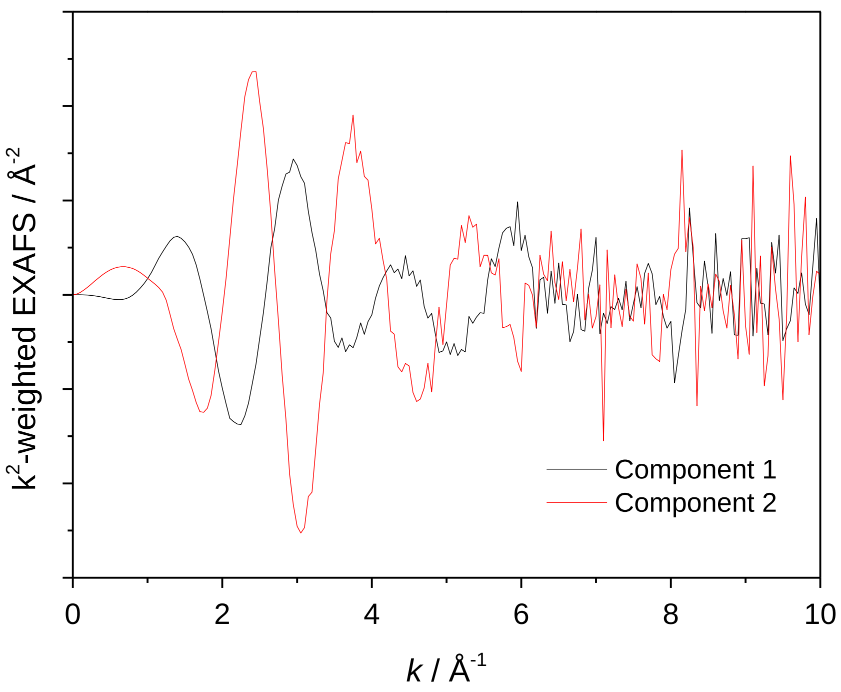

Figure S5: Background extracted k^2-weighted EXAFS data of individual Cs K spectral components extracted using MCR-ALS.

SI-5: Concentrations of Ru and Cs species during ammonia synthesis at higher temperatures

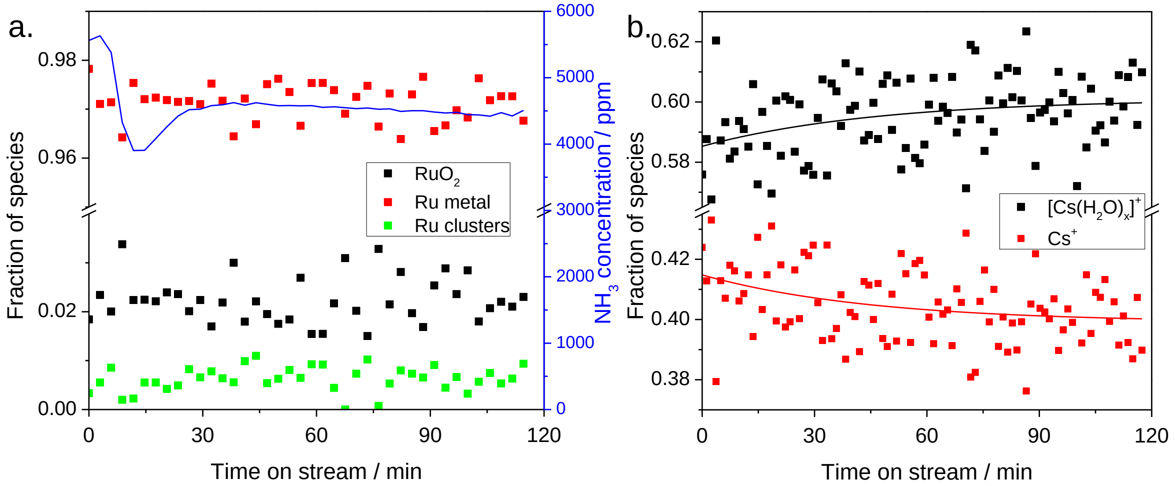

Figure S6: Evolution of fractions of (a.) Ru and (b.) Cs species measured during ammonia synthesis in H_2:N_2 = 3:1 feed with 25 ppm O_2 at 490 °C for the RuCs/MgO catalyst. Blue curves stand for produced ammonia (a drop in the beginning is due to the large dead volume of the setup and the previous step being heating from 386 °C to 490 °C).

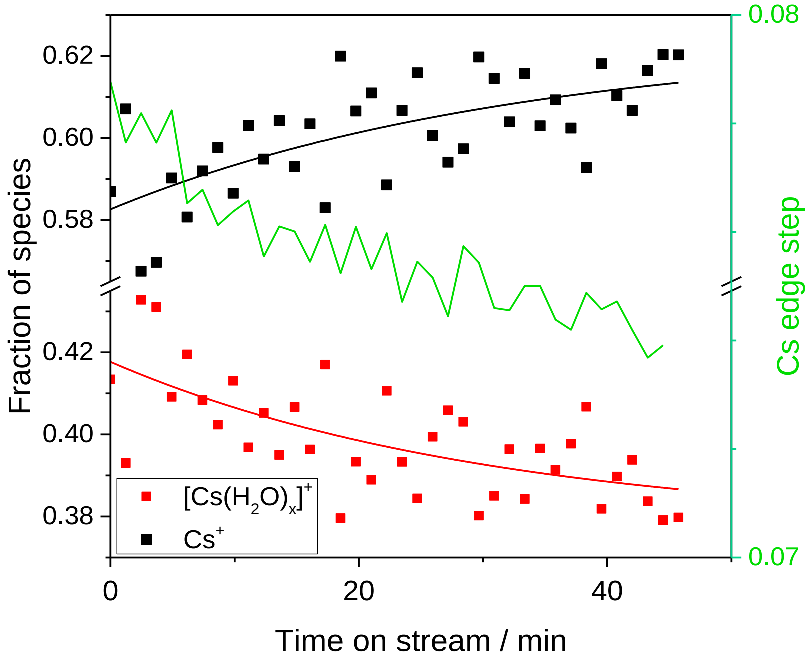

Figure S7: Evolution of fractions of Cs species and Cs edge step (Ru was not recorded) measured during ammonia synthesis in H_2:N_2 = 3:1 feed with 25 ppm O_2 at 686 °C for the RuCs/MgO catalyst.

SI-6: Calculated temperature dependence of equilibrium concentrations of Cs cations coordinated with 1 to 4 water ligands

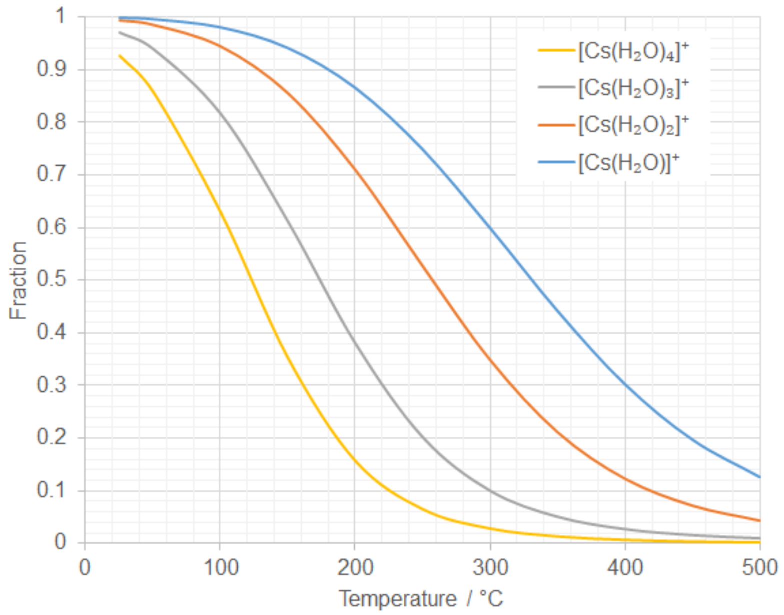

Figure S8: Equilibrium concentrations of $[Cs(H_2O)_x]^+$ during its thermal decomposition with loss of one water ligand, where *x = 1 – 4*. Calculation is based on the data from ref. [2].

SI-7: Ru K edge XANES, EXAFS spectra of the Ru/MgO and RuCs/MgO, and the corresponding EXAFS analysis

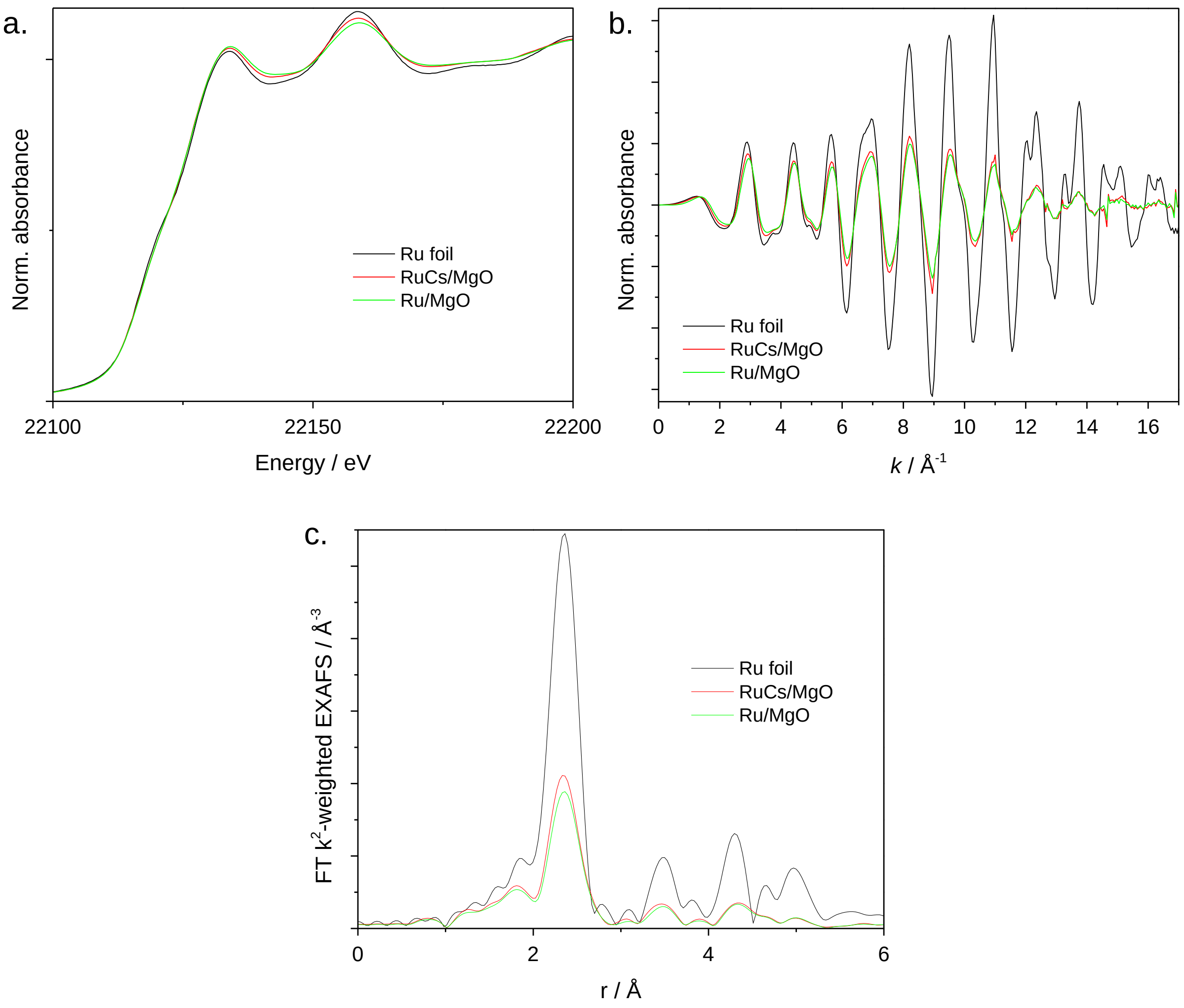

Figure S9: *Operando* Ru K edge (a) XANES, (b) k^2-weighted EXAFS, and (c) FT k^2-weighted EXAFS spectra (k-range 2.5 – 16.5 Å^{-1}) of the Ru/MgO and RuCs/MgO catalysts measured during ammonia synthesis in pure H_2:N_2 = 3:1 feed at 386 °C (average of all spectra recorded under unchanging conditions). *Ex situ* Ru foil spectrum (at 20 °C) is provided for a comparison.

Table S1: EXAFS first shell fitting results for the Ru/MgO and RuCs/MgO catalysts measured during ammonia synthesis in pure H_2:N_2 = 3:1 feed at 386 °C. *k*-range: 2.5 – 16.5 Å^{-1}, R-range: 1.5 – 2.7 Å, S_0^2 = 0.76±0.7.

Sample	*Ru^0 fraction**	C*N*	*R (Å)*	*σ^2 (Å^2)*	*ΔE_0 (eV)*	*ρ*	C*N* corr.**
Ru foil	1	12***	2.672(4)	0.0036(4)	-6.9(8)	0.014	12
Ru/MgO	0.948	8.5(5)	2.662(4)	0.0075(5)	-6.6(5)	0.011	9.0
RuCs/MgO	0.973	9.4(6)	2.662(4)	0.0074(5)	-7.4(5)	0.011	9.7

* Based on the weight determined from MCR-ALS.
** Corrected to exclude contribution of (partially) oxidized Ru.
*** Value was fixed in order to obtain the amplitude reduction factor.

References:

[1] J. J. Kas, F. D. Vila, C. D. Pemmaraju, T. S. Tan, J. J. Rehr, *J Synchrotron Rad* **2021**, *28*, 1801–1810.
[2] I. Dzidic, P. Kebarle, *J. Phys. Chem.* **1970**, *74*, 1466–1474.

SI-8: FEFF.inp input files for the XANES calculations

```
TITLE RuO2

EDGE K
S02 1.0

CONTROL 1 1 1 1 1 1

PRINT 1 0 0 0 0 0

EXCHANGE 0 0.0 0.0 -1
SCF 5.0 0 100 0.2 1
COREHOLE NONE
LDOS -40 30 0.1
* RSIGMA
FMS 6.0 0
XANES 5 0.07 0

POTENTIALS
0 44 Ru
1 44 Ru
2 8 O


ATOMS
 0.00000   0.00000   0.00000  0 Ru1      0.00000
 0.00000   1.94260   0.00000  2 O1.1     1.94260
 0.00000  -1.94260   0.00000  2 O1.1     1.94260
 1.23366   0.00000   1.55330  2 O1.2     1.98360
-1.23366   0.00000   1.55330  2 O1.2     1.98360
 1.23366   0.00000  -1.55330  2 O1.2     1.98360
-1.23366   0.00000  -1.55330  2 O1.2     1.98360
 0.00000   0.00000   3.10660  1 Ru1.1    3.10660
 0.00000   0.00000  -3.10660  1 Ru1.1    3.10660
 3.17625   1.23366   0.00000  2 O1.3     3.40742
-3.17625   1.23366   0.00000  2 O1.3     3.40742
 3.17625  -1.23366   0.00000  2 O1.3     3.40742
-3.17625  -1.23366   0.00000  2 O1.3     3.40742
 3.17625   0.00000   1.55330  1 Ru1.2    3.53572
-3.17625   0.00000   1.55330  1 Ru1.2    3.53572
 0.00000   3.17625   1.55330  1 Ru1.2    3.53572
 0.00000  -3.17625   1.55330  1 Ru1.2    3.53572
 3.17625   0.00000  -1.55330  1 Ru1.2    3.53572
-3.17625   0.00000  -1.55330  1 Ru1.2    3.53572
 0.00000   3.17625  -1.55330  1 Ru1.2    3.53572
 0.00000  -3.17625  -1.55330  1 Ru1.2    3.53572
 0.00000   1.94260   3.10660  2 O1.4     3.66397
 0.00000  -1.94260   3.10660  2 O1.4     3.66397
 0.00000   1.94260  -3.10660  2 O1.4     3.66397
 0.00000  -1.94260  -3.10660  2 O1.4     3.66397
 1.94260   3.17625   1.55330  2 O1.5     4.03423
-1.94260   3.17625   1.55330  2 O1.5     4.03423
 1.94260  -3.17625   1.55330  2 O1.5     4.03423
-1.94260  -3.17625   1.55330  2 O1.5     4.03423
 1.94260   3.17625  -1.55330  2 O1.5     4.03423
-1.94260   3.17625  -1.55330  2 O1.5     4.03423
 1.94260  -3.17625  -1.55330  2 O1.5     4.03423
-1.94260  -3.17625  -1.55330  2 O1.5     4.03423
 0.00000   4.40991   0.00000  2 O1.6     4.40991
 0.00000  -4.40991   0.00000  2 O1.6     4.40991
 3.17625   3.17625   0.00000  1 Ru1.3    4.49190
-3.17625   3.17625   0.00000  1 Ru1.3    4.49190
 3.17625  -3.17625   0.00000  1 Ru1.3    4.49190
-3.17625  -3.17625   0.00000  1 Ru1.3    4.49190
 3.17625   1.23366   3.10660  2 O1.7     4.61102
-3.17625   1.23366   3.10660  2 O1.7     4.61102
 3.17625  -1.23366   3.10660  2 O1.7     4.61102
-3.17625  -1.23366   3.10660  2 O1.7     4.61102
 3.17625   1.23366  -3.10660  2 O1.7     4.61102
-3.17625   1.23366  -3.10660  2 O1.7     4.61102
 3.17625  -1.23366  -3.10660  2 O1.7     4.61102
-3.17625  -1.23366  -3.10660  2 O1.7     4.61102
 1.23366   0.00000   4.65990  2 O1.8     4.82043
-1.23366   0.00000   4.65990  2 O1.8     4.82043
 1.23366   0.00000  -4.65990  2 O1.8     4.82043
-1.23366   0.00000  -4.65990  2 O1.8     4.82043
 5.11885   0.00000   1.55330  2 O1.9     5.34933
-5.11885   0.00000   1.55330  2 O1.9     5.34933
 5.11885   0.00000  -1.55330  2 O1.9     5.34933
```

-5.11885	0.00000	-1.55330	2 O1.9	5.34933
0.00000	4.40991	3.10660	2 O1.10	5.39428
0.00000	-4.40991	3.10660	2 O1.10	5.39428
0.00000	4.40991	-3.10660	2 O1.10	5.39428
0.00000	-4.40991	-3.10660	2 O1.10	5.39428
3.17625	3.17625	3.10660	1 Ru1.4	5.46151
-3.17625	3.17625	3.10660	1 Ru1.4	5.46151
3.17625	-3.17625	3.10660	1 Ru1.4	5.46151
-3.17625	-3.17625	3.10660	1 Ru1.4	5.46151
3.17625	3.17625	-3.10660	1 Ru1.4	5.46151
-3.17625	3.17625	-3.10660	1 Ru1.4	5.46151
3.17625	-3.17625	-3.10660	1 Ru1.4	5.46151
-3.17625	-3.17625	-3.10660	1 Ru1.4	5.46151
3.17625	0.00000	4.65990	1 Ru1.5	5.63944
-3.17625	0.00000	4.65990	1 Ru1.5	5.63944
0.00000	3.17625	4.65990	1 Ru1.5	5.63944
0.00000	-3.17625	4.65990	1 Ru1.5	5.63944
3.17625	0.00000	-4.65990	1 Ru1.5	5.63944
-3.17625	0.00000	-4.65990	1 Ru1.5	5.63944
0.00000	3.17625	-4.65990	1 Ru1.5	5.63944
0.00000	-3.17625	-4.65990	1 Ru1.5	5.63944
4.40991	3.17625	1.55330	2 O1.11	5.65231
-4.40991	3.17625	1.55330	2 O1.11	5.65231
4.40991	-3.17625	1.55330	2 O1.11	5.65231
-4.40991	-3.17625	1.55330	2 O1.11	5.65231
4.40991	3.17625	-1.55330	2 O1.11	5.65231
-4.40991	3.17625	-1.55330	2 O1.11	5.65231
4.40991	-3.17625	-1.55330	2 O1.11	5.65231
-4.40991	-3.17625	-1.55330	2 O1.11	5.65231
1.94260	3.17625	4.65990	2 O1.12	5.96464
-1.94260	3.17625	4.65990	2 O1.12	5.96464
1.94260	-3.17625	4.65990	2 O1.12	5.96464
-1.94260	-3.17625	4.65990	2 O1.12	5.96464
1.94260	3.17625	-4.65990	2 O1.12	5.96464
-1.94260	3.17625	-4.65990	2 O1.12	5.96464
1.94260	-3.17625	-4.65990	2 O1.12	5.96464
-1.94260	-3.17625	-4.65990	2 O1.12	5.96464
3.17625	5.11885	0.00000	2 O1.13	6.02422
-3.17625	5.11885	0.00000	2 O1.13	6.02422
3.17625	-5.11885	0.00000	2 O1.13	6.02422
-3.17625	-5.11885	0.00000	2 O1.13	6.02422
0.00000	0.00000	6.21320	1 Ru1.6	6.21320
0.00000	0.00000	-6.21320	1 Ru1.6	6.21320
6.35251	0.00000	0.00000	1 Ru1.7	6.35251
-6.35251	0.00000	0.00000	1 Ru1.7	6.35251
0.00000	6.35251	0.00000	1 Ru1.7	6.35251
0.00000	-6.35251	0.00000	1 Ru1.7	6.35251
0.00000	1.94260	6.21320	2 O1.14	6.50980
0.00000	-1.94260	6.21320	2 O1.14	6.50980
0.00000	1.94260	-6.21320	2 O1.14	6.50980
0.00000	-1.94260	-6.21320	2 O1.14	6.50980
6.35251	1.94260	0.00000	2 O1.15	6.64289
-6.35251	1.94260	0.00000	2 O1.15	6.64289
6.35251	-1.94260	0.00000	2 O1.15	6.64289
-6.35251	-1.94260	0.00000	2 O1.15	6.64289
1.23366	6.35251	1.55330	2 O1.16	6.65500
-1.23366	6.35251	1.55330	2 O1.16	6.65500
1.23366	-6.35251	1.55330	2 O1.16	6.65500
-1.23366	-6.35251	1.55330	2 O1.16	6.65500
1.23366	6.35251	-1.55330	2 O1.16	6.65500
-1.23366	6.35251	-1.55330	2 O1.16	6.65500
1.23366	-6.35251	-1.55330	2 O1.16	6.65500
-1.23366	-6.35251	-1.55330	2 O1.16	6.65500
3.17625	5.11885	3.10660	2 O1.17	6.77806
-3.17625	5.11885	3.10660	2 O1.17	6.77806
3.17625	-5.11885	3.10660	2 O1.17	6.77806
-3.17625	-5.11885	3.10660	2 O1.17	6.77806
3.17625	5.11885	-3.10660	2 O1.17	6.77806
-3.17625	5.11885	-3.10660	2 O1.17	6.77806
3.17625	-5.11885	-3.10660	2 O1.17	6.77806
-3.17625	-5.11885	-3.10660	2 O1.17	6.77806
5.11885	0.00000	4.65990	2 O1.18	6.92223
-5.11885	0.00000	4.65990	2 O1.18	6.92223
5.11885	0.00000	-4.65990	2 O1.18	6.92223
-5.11885	0.00000	-4.65990	2 O1.18	6.92223
6.35251	0.00000	3.10660	1 Ru1.8	7.07144
-6.35251	0.00000	3.10660	1 Ru1.8	7.07144
0.00000	6.35251	3.10660	1 Ru1.8	7.07144
0.00000	-6.35251	3.10660	1 Ru1.8	7.07144
6.35251	0.00000	-3.10660	1 Ru1.8	7.07144
-6.35251	0.00000	-3.10660	1 Ru1.8	7.07144

```
 0.00000   6.35251  -3.10660   1 Ru1.8         7.07144
 0.00000  -6.35251  -3.10660   1 Ru1.8         7.07144
 3.17625   1.23366   6.21320   2 O1.19         7.08621
-3.17625   1.23366   6.21320   2 O1.19         7.08621
 3.17625  -1.23366   6.21320   2 O1.19         7.08621
-3.17625  -1.23366   6.21320   2 O1.19         7.08621
 3.17625   1.23366  -6.21320   2 O1.19         7.08621
-3.17625   1.23366  -6.21320   2 O1.19         7.08621
 3.17625  -1.23366  -6.21320   2 O1.19         7.08621
-3.17625  -1.23366  -6.21320   2 O1.19         7.08621
 4.40991   3.17625   4.65990   2 O1.20         7.15895
-4.40991   3.17625   4.65990   2 O1.20         7.15895
 4.40991  -3.17625   4.65990   2 O1.20         7.15895
-4.40991  -3.17625   4.65990   2 O1.20         7.15895
 4.40991   3.17625  -4.65990   2 O1.20         7.15895
-4.40991   3.17625  -4.65990   2 O1.20         7.15895
 4.40991  -3.17625  -4.65990   2 O1.20         7.15895
-4.40991  -3.17625  -4.65990   2 O1.20         7.15895
 6.35251   3.17625   1.55330   1 Ru1.9         7.27019
-6.35251   3.17625   1.55330   1 Ru1.9         7.27019
 3.17625   6.35251   1.55330   1 Ru1.9         7.27019
-3.17625   6.35251   1.55330   1 Ru1.9         7.27019
 6.35251  -3.17625   1.55330   1 Ru1.9         7.27019
-6.35251  -3.17625   1.55330   1 Ru1.9         7.27019
 3.17625  -6.35251   1.55330   1 Ru1.9         7.27019
-3.17625  -6.35251   1.55330   1 Ru1.9         7.27019
 6.35251   3.17625  -1.55330   1 Ru1.9         7.27019
-6.35251   3.17625  -1.55330   1 Ru1.9         7.27019
 3.17625   6.35251  -1.55330   1 Ru1.9         7.27019
-3.17625   6.35251  -1.55330   1 Ru1.9         7.27019
 6.35251  -3.17625  -1.55330   1 Ru1.9         7.27019
-6.35251  -3.17625  -1.55330   1 Ru1.9         7.27019
 3.17625  -6.35251  -1.55330   1 Ru1.9         7.27019
-3.17625  -6.35251  -1.55330   1 Ru1.9         7.27019
 6.35251   1.94260   3.10660   2 O1.21         7.33342
-6.35251   1.94260   3.10660   2 O1.21         7.33342
 6.35251  -1.94260   3.10660   2 O1.21         7.33342
-6.35251  -1.94260   3.10660   2 O1.21         7.33342
 6.35251   1.94260  -3.10660   2 O1.21         7.33342
-6.35251   1.94260  -3.10660   2 O1.21         7.33342
 6.35251  -1.94260  -3.10660   2 O1.21         7.33342
-6.35251  -1.94260  -3.10660   2 O1.21         7.33342
 0.00000   4.40991   6.21320   2 O1.22         7.61913
 0.00000  -4.40991   6.21320   2 O1.22         7.61913
 0.00000   4.40991  -6.21320   2 O1.22         7.61913
 0.00000  -4.40991  -6.21320   2 O1.22         7.61913

END
```

```
TITLE Ruthenium
TITLE Ru

EDGE K
S02 1.0

CONTROL 1 1 1 1 1 1

PRINT 1 0 0 0 0 0

EXCHANGE 0 0.0 0.0 -1
SCF 5.0 0 100 0.2 1
COREHOLE NONE
LDOS -40 30 0.1
* RSIGMA
FMS 6.0 0
XANES 5 0.07 0

POTENTIALS
0 44 Ru
1 44 Ru


ATOMS
 0.00000   0.00000   0.00000   0 Ru1           0.00000
-0.78110  -1.35281   2.14055   1 Ru1.1         2.64994
-0.78110  -1.35281  -2.14055   1 Ru1.1         2.64994
 1.56219   0.00009   2.14055   1 Ru1.1         2.64998
 1.56219   0.00009  -2.14055   1 Ru1.1         2.64998
-0.78110   1.35299   2.14055   1 Ru1.1         2.65003
-0.78110   1.35299  -2.14055   1 Ru1.1         2.65003
```

2.34329	1.35290	0.00000	1 Ru1.2	2.70580
-2.34329	1.35290	0.00000	1 Ru1.2	2.70580
0.00000	2.70580	0.00000	1 Ru1.2	2.70580
2.34329	-1.35290	0.00000	1 Ru1.2	2.70580
-2.34329	-1.35290	0.00000	1 Ru1.2	2.70580
0.00000	-2.70580	0.00000	1 Ru1.2	2.70580
1.56219	-2.70571	2.14055	1 Ru1.3	3.78725
1.56219	-2.70571	-2.14055	1 Ru1.3	3.78725
-3.12439	0.00009	2.14055	1 Ru1.3	3.78732
-3.12439	0.00009	-2.14055	1 Ru1.3	3.78732
1.56219	2.70589	2.14055	1 Ru1.3	3.78738
1.56219	2.70589	-2.14055	1 Ru1.3	3.78738
0.00000	0.00000	4.28110	1 Ru1.4	4.28110
0.00000	0.00000	-4.28110	1 Ru1.4	4.28110
-0.78110	-4.05861	2.14055	1 Ru1.5	4.65450
-0.78110	-4.05861	-2.14055	1 Ru1.5	4.65450
-3.12439	-2.70571	2.14055	1 Ru1.5	4.65453
-3.12439	-2.70571	-2.14055	1 Ru1.5	4.65453
3.90549	-1.35281	2.14055	1 Ru1.5	4.65455
3.90549	-1.35281	-2.14055	1 Ru1.5	4.65455
3.90549	1.35299	2.14055	1 Ru1.5	4.65461
3.90549	1.35299	-2.14055	1 Ru1.5	4.65461
-3.12439	2.70589	2.14055	1 Ru1.5	4.65463
-3.12439	2.70589	-2.14055	1 Ru1.5	4.65463
-0.78110	4.05879	2.14055	1 Ru1.5	4.65466
-0.78110	4.05879	-2.14055	1 Ru1.5	4.65466
4.68658	0.00000	0.00000	1 Ru1.6	4.68658
-4.68658	0.00000	0.00000	1 Ru1.6	4.68658
2.34329	4.05870	0.00000	1 Ru1.6	4.68658
-2.34329	4.05870	0.00000	1 Ru1.6	4.68658
2.34329	-4.05870	0.00000	1 Ru1.6	4.68658
-2.34329	-4.05870	0.00000	1 Ru1.6	4.68658
2.34329	1.35290	4.28110	1 Ru1.7	5.06450
-2.34329	1.35290	4.28110	1 Ru1.7	5.06450
0.00000	2.70580	4.28110	1 Ru1.7	5.06450
2.34329	-1.35290	4.28110	1 Ru1.7	5.06450
-2.34329	-1.35290	4.28110	1 Ru1.7	5.06450
0.00000	-2.70580	4.28110	1 Ru1.7	5.06450
2.34329	1.35290	-4.28110	1 Ru1.7	5.06450
-2.34329	1.35290	-4.28110	1 Ru1.7	5.06450
0.00000	2.70580	-4.28110	1 Ru1.7	5.06450
2.34329	-1.35290	-4.28110	1 Ru1.7	5.06450
-2.34329	-1.35290	-4.28110	1 Ru1.7	5.06450
0.00000	-2.70580	-4.28110	1 Ru1.7	5.06450
4.68658	2.70580	0.00000	1 Ru1.8	5.41160
-4.68658	2.70580	0.00000	1 Ru1.8	5.41160
0.00000	5.41160	0.00000	1 Ru1.8	5.41160
4.68658	-2.70580	0.00000	1 Ru1.8	5.41160
-4.68658	-2.70580	0.00000	1 Ru1.8	5.41160
0.00000	-5.41160	0.00000	1 Ru1.8	5.41160
1.56219	-5.41151	2.14055	1 Ru1.9	6.02552
1.56219	-5.41151	-2.14055	1 Ru1.9	6.02552
3.90549	-4.05861	2.14055	1 Ru1.9	6.02554
3.90549	-4.05861	-2.14055	1 Ru1.9	6.02554
-5.46768	-1.35281	2.14055	1 Ru1.9	6.02558
-5.46768	-1.35281	-2.14055	1 Ru1.9	6.02558
-5.46768	1.35299	2.14055	1 Ru1.9	6.02562
-5.46768	1.35299	-2.14055	1 Ru1.9	6.02562
3.90549	4.05879	2.14055	1 Ru1.9	6.02566
3.90549	4.05879	-2.14055	1 Ru1.9	6.02566
1.56219	5.41169	2.14055	1 Ru1.9	6.02568
1.56219	5.41169	-2.14055	1 Ru1.9	6.02568
4.68658	0.00000	4.28110	1 Ru1.10	6.34759
-4.68658	0.00000	4.28110	1 Ru1.10	6.34759
2.34329	4.05870	4.28110	1 Ru1.10	6.34759
-2.34329	4.05870	4.28110	1 Ru1.10	6.34759
2.34329	-4.05870	4.28110	1 Ru1.10	6.34759
-2.34329	-4.05870	4.28110	1 Ru1.10	6.34759
4.68658	0.00000	-4.28110	1 Ru1.10	6.34759
-4.68658	0.00000	-4.28110	1 Ru1.10	6.34759
2.34329	4.05870	-4.28110	1 Ru1.10	6.34759
-2.34329	4.05870	-4.28110	1 Ru1.10	6.34759
2.34329	-4.05870	-4.28110	1 Ru1.10	6.34759
-2.34329	-4.05870	-4.28110	1 Ru1.10	6.34759
-3.12439	-5.41151	2.14055	1 Ru1.11	6.60516
-3.12439	-5.41151	-2.14055	1 Ru1.11	6.60516
6.24878	0.00009	2.14055	1 Ru1.11	6.60524
6.24878	0.00009	-2.14055	1 Ru1.11	6.60524
-3.12439	5.41169	2.14055	1 Ru1.11	6.60531
-3.12439	5.41169	-2.14055	1 Ru1.11	6.60531
-0.78110	-1.35281	6.42165	1 Ru1.12	6.60892

```
 -0.78110   -1.35281   -6.42165    1 Ru1.12          6.60892
  1.56219    0.00009    6.42165    1 Ru1.12          6.60894
  1.56219    0.00009   -6.42165    1 Ru1.12          6.60894
 -0.78110    1.35299    6.42165    1 Ru1.12          6.60895
 -0.78110    1.35299   -6.42165    1 Ru1.12          6.60895
  4.68658    2.70580    4.28110    1 Ru1.13          6.90023
 -4.68658    2.70580    4.28110    1 Ru1.13          6.90023
  0.00000    5.41160    4.28110    1 Ru1.13          6.90023
  4.68658   -2.70580    4.28110    1 Ru1.13          6.90023
 -4.68658   -2.70580    4.28110    1 Ru1.13          6.90023
  0.00000   -5.41160    4.28110    1 Ru1.13          6.90023
  4.68658    2.70580   -4.28110    1 Ru1.13          6.90023
 -4.68658    2.70580   -4.28110    1 Ru1.13          6.90023
  0.00000    5.41160   -4.28110    1 Ru1.13          6.90023
  4.68658   -2.70580   -4.28110    1 Ru1.13          6.90023
 -4.68658   -2.70580   -4.28110    1 Ru1.13          6.90023
  0.00000   -5.41160   -4.28110    1 Ru1.13          6.90023

END
```

```
TITLE Ru4O4 cluster

EDGE K
S02 1.0

CONTROL 1 1 1 1 1 1

PRINT 1 0 0 0 0 0

EXCHANGE 0 0.0 0.0 -1
SCF 5.0 0 100 0.2 1
COREHOLE NONE
LDOS -40 30 0.1
* RSIGMA
FMS 6.0 0
XANES 5 0.07 0

POTENTIALS
0 44 Ru
1 44 Ru
2 8 O


ATOMS
  0.12901    1.79252    4.87440    0
  2.93058    1.70420    5.83548    1
  1.50083    4.29453    5.67338    1
  2.30769    2.88980    3.19225    1
  4.60567    4.26688    4.92930    2
 -0.49046    4.42755    3.18110    2
  2.11031   -0.28430    3.47595    2
  0.64260    2.27093    7.98916    2

END
```

```
TITLE Ru_ox_surface

EDGE K
S02 1.0

CONTROL 1 1 1 1 1 1

PRINT 1 0 0 0 0 0

EXCHANGE 0 0.0 0.0 -1
SCF 5.0 0 100 0.2 1
COREHOLE NONE
LDOS -40 30 0.1
* RSIGMA
FMS 6.0 0
XANES 5 0.07 0

POTENTIALS
0 44 Ru
1 44 Ru
2 8 O


ATOMS
```

-0.00838	0.06152	-0.00196	1
-0.86373	-1.65322	1.70278	1
-0.95384	-1.79717	-1.78733	1
2.07507	-0.08140	1.74989	1
2.05863	0.00562	-1.83488	1
-0.99514	2.01672	1.80244	1
-0.71917	1.76287	-1.77000	1
2.93837	1.77506	-0.05784	1
-2.90675	1.79484	-0.07965	1
-0.00745	3.90306	-0.02451	1
2.94863	-1.98579	-0.05894	1
-3.10513	-1.92272	-0.13173	1
-0.31047	-3.74866	-0.03361	1
1.76827	-3.80485	1.94086	1
1.81512	-3.68159	-2.03318	1
-4.10301	-0.16052	1.69795	1
-3.99258	0.10656	-2.03307	1
2.07506	3.54338	1.83568	1
2.08926	3.98854	-1.87416	1
0.03649	0.25276	3.59067	1
0.02066	-0.12133	-3.54608	1
-0.93880	-5.77793	1.81273	1
-0.89764	-5.81824	-1.82129	1
-3.87682	-3.84810	1.75565	1
-4.03075	-3.98469	-1.90670	1
4.90769	-1.57838	1.81405	1
5.35772	-1.97341	-1.79753	1
5.33466	2.06400	1.69081	1
4.87578	1.72256	-1.96925	1
-3.79417	3.59075	1.81756	1
-3.86586	3.87242	-1.84547	1
-0.86795	5.88557	1.72886	1
-1.17275	5.47735	-1.90731	1
6.43603	0.09880	-0.09119	1
-6.29290	0.26877	0.01606	1
3.66260	5.39725	0.04609	1
-3.32989	5.88313	0.08024	1
2.91652	-5.71966	0.08477	1
-3.22439	-6.06525	-0.01557	1
3.31468	1.57119	3.48153	1
-3.06783	1.48631	3.51674	1
-0.09608	3.77416	3.68280	1
2.72129	-1.80930	3.59962	1
-2.77949	-1.97164	3.32363	1
-0.63820	-3.56693	3.71123	1
2.51134	2.02130	-3.63980	1
-2.66760	2.18096	-3.65302	1
-0.11349	3.43252	-3.70949	1
3.22415	-1.62574	-3.67119	1
-2.87661	-1.91443	-3.60902	1
-0.61046	-3.68861	-3.81586	1
5.30870	4.20357	-0.13290	1
-6.00880	2.92341	-0.00140	1
0.26096	7.59905	-0.21350	1
5.82330	-3.98264	0.16265	1
-6.17634	-3.19282	0.02211	1
0.84039	-7.13130	0.04531	1
2.71358	-7.78954	2.21328	1
2.70266	-7.74935	-2.13844	1
5.07116	-5.93760	2.22451	1
4.98592	-5.89781	-2.01644	1
-7.43812	-1.48143	2.21097	1
-7.16656	-1.45125	-2.16297	1
-7.52918	1.75357	2.27704	1
-7.47169	1.58392	-2.34313	1
5.14762	6.17416	2.36127	1
5.19918	5.99198	-2.49519	1
2.39213	7.53637	1.85950	1
2.54695	7.25926	-1.96325	1
5.83056	0.16639	3.97169	1
-5.61223	0.15704	4.07433	1
2.46996	5.38023	3.98825	1
-2.60976	5.34366	3.96537	1
2.69470	-5.31386	4.14432	1
-2.80339	-5.42985	4.00086	1
5.73716	-0.14557	-4.12042	1
-5.70671	0.06193	-4.33457	1
2.82842	5.41689	-4.28277	1
-3.19923	5.83670	-4.03841	1
2.77327	-5.41527	-4.14131	1
-2.79253	-5.54516	-3.98111	1

-4.34593	-7.54824	2.39729	1
-4.24942	-7.70921	-2.35475	1
8.36588	-0.19242	2.24296	1
8.32106	0.27341	-2.48606	1
-4.33714	7.38435	2.51583	1
-4.23009	7.86325	-2.04124	1
-0.98812	-1.27805	5.70588	1
-0.90123	-1.45715	-5.67753	1
1.76662	0.14261	5.56251	0
1.82905	-0.03382	-5.66124	1
-0.97498	1.76818	5.56202	1
-0.75001	1.41774	-5.85803	1
5.16874	3.55494	4.22776	1
-5.29130	3.20018	4.17997	1
0.05752	7.33481	3.89883	1
5.04439	-3.25388	4.13858	1
-5.20479	-2.93530	4.19590	1
0.10532	-7.09153	4.02297	1
4.93466	3.26171	-4.38611	1
-5.27430	3.08800	-4.26057	1
0.07958	6.76176	-4.06830	1
5.21771	-3.54951	-4.23764	1
-5.23616	-3.00983	-4.36862	1
0.16469	-7.16890	-4.04114	1
4.34906	-0.72335	6.15101	2
1.76496	2.84894	5.38167	2
-3.46630	-0.21709	5.73050	2

END
